# Medical Department of the University of Buffalo

**Published:** 1870-04

**Authors:** 


### Medical Department of the University of Buffalo.

At a regular meeting of the Faculty of the Medical Department of the University of Buffalo, Prof. Charles A. Lee, was appointed delegate to the National Convention for revising the Pharmacopœia to be held in Washington the first Monday of May next.

